# The potential of [^18^F]FDG PET/MRI for identifying pain sources in patients with diagnostically challenging chronic hip pain after total hip replacement

**DOI:** 10.1007/s00259-026-08142-4

**Published:** 2026-08-22

**Authors:** Jacob M. Mostert, Pieter K. Bos, Michael Ananta, Galied S. R. Muradin, Sandip Biswal, Edwin H.G. Oei, Rianne A. van der Heijden

**Affiliations:** 1 Department of Radiology & Nuclear Medicine, Erasmus University Medical Center, Rotterdam, The Netherlands; 2 Department of Orthopaedics & Sports Medicine, Erasmus University Medical Center, Rotterdam, The Netherlands; 3 Department of Radiology, University of Wisconsin-Madison, Madison, WI USA

**Keywords:** Pain, PET/MRI, Joint arthroplasty, Hip

## Abstract

**Purpose:**

Chronic hip pain after total hip replacement (THR) poses a diagnostic challenge and frequently yields lengthy and unsatisfactory diagnostic work-up. This study aims to evaluate the diagnostic role and clinical impact of [^18^F]FDG PET/MRI compared with regular diagnostic work-up in patients with chronic hip pain after THR.

**Methods:**

In this randomized study, 20 patients with persistent hip pain of unclear origin after THR underwent either PET/MRI-guided clinical management or regular diagnostic work-up. Additional diagnostic and therapeutic procedures during 12-month follow-up were recorded and a final expert diagnosis was determined through expert opinion based on all available information. Patient reported outcomes included pain scores, functional scores and quality of life.

**Results:**

In the PET/MRI group, a final expert diagnosis could be established for 8/10 patients compared to 5/10 in the control group. PET-MRI contributed to this diagnosis in 7 of 10 patients by identifying 5 new pain sources, confirming 3 clinically suspected pain sources, and excluding 1 clinically suspected pain source. At 6 months, mean pain scores on NRS 0–10 decreased in both groups (PET/MRI: 6.1 to 5.2; control: 5.2 to 3.4). Between-group differences could not be statistically tested due to insufficient statistical power.

**Conclusion:**

Initial results demonstrate the potential of [^18^F]FDG PET/MRI for the identification of pain sources in patients with diagnostically challenging chronic hip pain after THR. Further research regarding clinical outcome is warranted in a larger cohort with standardized treatment strategies incorporating PET/MRI findings.

**Supplementary Information:**

The online version contains supplementary material available at https://doi.org/10.1007/s00259-026-08142-4.

## Introduction

Total hip replacement (THR) is a common surgical intervention that can restore function and relieve pain in patients with severe hip abnormalities. In Europe alone, over 1 million THR surgeries are performed each year [1]. However, between 8.5% and 20.1% of patients experience long-term hip pain after THR [2, 3]. As the number of hip arthroplasties is predicted to significantly increase over the next decades, the number of patients presenting with persisting pain will rise proportionally [4]. The diagnostic work-up and clinical management of chronic hip pain in these patients is often challenging [5]. Implant infection and aseptic loosening are common causes of pain after THR, and may be diagnosed by radiography and synovial fluid cultures, accompanied by blood laboratory analysis [6, 7]. When diagnostic tests for infection are negative, the likelihood of an infection is low, and other potential causes should be considered. Other possible pain causes then include soft tissue irritation such as tendinopathy or bursitis, mechanical impingement by implant components or bone, adverse local tissue reactions, neurological or vascular causes, or referred pain from the spine [5, 8]. Clinical examination and radiography alone cannot differentiate between this multitude of potential pain generators, necessitating additional diagnostic and therapeutic interventions. The diagnostic work-up to identify the pain source in these patients can consist of multiple imaging modalities including ultrasonography, CT and MRI, as well as molecular imaging such as bone scintigraphy and sometimes positron emission tomography (PET) [8–10]. Additionally, injection of anaesthetics can also have a role, both as a diagnostic and therapeutic modality [8].

All of these diagnostic modalities may have a role in the work-up and management of chronic post-THR pain. However, the consecutive application of these different modalities is expensive and leads to a lengthy diagnostic work-up. Moreover, it does not always lead to the identification of the actual source of pain, especially since the distinction between pain generators and pre-existent post-surgical changes further complicates the picture [5]. Hybrid PET/MRI may be a promising alternative, by providing combined functional and anatomical imaging in a single imaging session. PET imaging with the radiolabelled glucose analogue fluorodeoxyglucose ([^18^F]FDG) provides information on tissue glucose metabolism and may identify low-grade inflammation occurring in the context of pain [11]. Combined with precise anatomical localization including soft tissue structural changes of MRI, its potential for detecting pain sources has been demonstrated in patients with chronic low back pain [12–16]. As such, [^18^F]FDG PET/MRI may also provide comprehensive diagnostic information in one imaging session for patients with diagnostically challenging chronic pain after THR.

In this study, we aim to evaluate the diagnostic role and clinical impact of [^18^F]FDG PET/MRI in patients with persistent hip pain of unclear origin after THR.

## Methods

### Study design

This was a single-center randomized study with a PET MRI group and a control group with regular diagnostic work-up without PET-MRI. All other diagnostic tests and therapies were neither randomized nor standardized, but followed routine clinical practice. The study was part of a two-part trial involving patients with low back pain and hip pain. The Erasmus MC institutional review board approved the study (MEC-2021-0431) and the study was registered in the Dutch clinical trial registry (NL-OMON54240**).** All participants provided written informed consent. Study participants were 1:1 block-randomized into either the intervention or control arm. Participants in the intervention group underwent [^18^F]FDG PET/MRI imaging followed by targeted treatment when indicated. Control participants did not undergo PET/MRI and received regular diagnostic work-up instead, with subsequent treatment when indicated. Due to the nature of the intervention, patients and treating physicians were not blinded to group allocation.

### Study population

Patients aged 18–75 years with persisting hip pain of unclear origin after ipsilateral THR were included. Patients were recruited in our tertiary care academic hospital orthopedic outpatient clinic, after being referred from community hospitals following unsatisfactory outcomes. Inclusion criteria were pain in the hip region for more than 3 months with unclear origin, ipsilateral THR more than 10 months ago, no radiographic signs of prosthetic joint infection or aseptic loosening, and a patient reported pain score of 3 or higher on a numeric rating scale (NRS) 0–10. Exclusion criteria were diagnosed cancer, pregnancy or breastfeeding, MRI non-compatible implants (e.g. implantable cardiac devices), severe claustrophobia, body mass index > 35 kg/m^2^, diabetes mellitus, known renal insufficiency, or known intolerance to gadolinium-based contrast agents.

### [^18^F]FDG PET/MRI acquisition and image analysis

Hybrid PET-MR imaging was performed on a clinical 3 Tesla PET/MRI system (SIGNA PET/MR, GE Healthcare, Waukesha, WI, USA). Patients fasted for at least six hours before intravenous administration of 0.033 MBq/kg^2^ [^18^F]FDG. Whole-body sequential PET acquisition started 60 min after tracer injection. PET acquisition time was set to 3 min per bed position, with 8 to 10 bed positions to cover the body from head to lower legs. Simultaneous T1-weighted and T2-weighted MR images with Dixon fat-water separation were acquired for each bed position. Dedicated T1-weighted Dixon images were acquired for PET attenuation correction.

Hybrid PET-MR imaging was supplemented with dedicated MR imaging of the pelvic region and lumbar spine, using multi-acquisition with variable resonance image combination and slice-selectivity (MAVRIC-SL) sequences to reduce in-plane and through-plane artefacts induced by the metal hip implants [17]. Contrast-enhanced images were acquired after intravenous administration of a single bolus of 0.1 mmol/kg gadobutrol. MRI pulse sequence parameters are presented in Supplementary Table S1.

PET data were corrected for random coincidences, dead time, scatter, and attenuation. Attenuation correction was performed by automated tissue classification on the dedicated MR images. Multiple PET image reconstructions were made using a block sequential regularized expectation maximization (BSREM) algorithm with penalty factor β set to 450 and 300 and one in accordance with the updated European Association of Nuclear Medicine guidelines (EARL) for PET quantification with bodyweight-corrected standardized uptake values (SUV) [18]. BSREM 450 was eventually used for clinical reading based on previous literature assessment [19] and reader consensus.

PET/MRI images were jointly evaluated by an experienced team consisting of one musculoskeletal radiologist (GM), one nuclear medicine physician with musculoskeletal affinity (MA) and a dual-trained musculoskeletal nuclear medicine radiologist with pain imaging experience (RH). When needed, a second MSK radiologist with PET pain imaging experience was consulted (SB). Potential pain generators were identified based on increased FDG uptake on PET and/or anatomical abnormalities or enhancement observed on MRI. Increased FDG uptake was defined as an elevated maximum standardized uptake value (SUV_max_) compared to contralateral structures and normal uptake patterns in asymptomatic hip implants [20, 21] and the lumbar spine [16, 22]. For periprosthetic FDG uptake, interpretation incorporated type of prosthesis, time since placement, and the specific uptake pattern [20], since physiological uptake may be seen up to 2 years after joint replacement [23]. All findings were reported to the treating orthopedic surgeons, who incorporated relevant findings in their treatment plans.

### Diagnostic work-up and interventions

For the participants in the PET/MRI group, the report and all images were accessible by the referring orthopedic surgeon. The radiologist could be consulted when needed. Participants randomized to the control group followed the diagnostic work-up as determined by the individual orthopedic surgeon. This diagnostic work-up was not standardized but tailored to each individual patient based on sign and symptom presentation and prior imaging, and could consist of conventional diagnostic imaging such as CT, MRI, or bone scintigraphy. Where appropriate, additional ultrasound imaging with or without diagnostic/therapeutic injection of local anesthetics and/or steroids was performed. In both groups, subsequent treatment was performed when indicated according to clinical guidelines.

### Study outcomes and follow-up

Although the cause of pain was unclear per the inclusion criteria, orthopedic surgeons were asked to provide a presumed diagnosis for study purposes based on medical history, clinical examination and available prior imaging. Study follow-up was 12 months for all participants. All suggested interventions based on PET/MRI and all performed interventions were recorded, including both diagnostic and therapeutic interventions. Short-term effects after interventions were collected from the patient record and were based on the patient and clinicians’ joint evaluation whether the treatment was successful. Treating physicians recorded their confidence in the diagnosis at baseline, as well as after PET/MRI imaging for participants in the intervention arm. Diagnostic confidence was rated on a standardized form with a 10-point probability score for each of the most prevalent causes of hip pain.

A final expert diagnosis for all participants was established by agreement of an expert orthopedic surgeon (PB) and an expert musculoskeletal radiologist (RH) at the end of the follow-up period based on all available diagnostic information and treatment outcomes. The added value of PET/MRI in the diagnostic process was categorized as follows: none, confirmed suspected diagnosis, excluded suspected diagnosis, or new pain source identified (confirmed during follow-up). Patient reported outcome measures (PROMs) were collected with questionnaires at baseline and at 1, 3, 6 and 12 months. PROMs included NRS pain score reported as the average pain score in the past week, worst NRS pain score in the past week, global perceived effect (GPE) and treatment satisfaction on 5-point Likert scales, quality of life using the EQ-5D-5 L questionnaire [24], and hip function using the Oxford Hip Score (OHS) [25]. EQ-5D-5 L responses were converted to a health state value using the Dutch national value set [26]. This provides a single EQ-5D-value that generally is between 0 and 1, although negative values are possible representing a health state considered worse than death. OHS consists of 12 questions with 5 possible answers, providing an overall OHS score ranging from 0 (worst) to 48 (best). At baseline, 6-month, and 12-month follow-up, participant surveys additionally included the hospital anxiety and depression scale (HADS) [27]. HADS questionnaires provided separate scores for anxiety and depression, both ranging from 0 to 21. A higher score indicates a higher severity of anxiety or depression.

### Analysis

Since the number of included participants was lower than anticipated and response to follow-up questionnaires was limited, the evaluation of study outcomes focuses on descriptive statistics. For both groups, the presumed diagnosis at baseline and final expert diagnosis after 12 months follow-up and all diagnostic and therapeutic procedures and treatment effects are listed. For the PET/MRI group, PET/MRI findings are listed and compared with the final expert diagnosis to determine the added value of FDG PET/MRI.

## Results

In total, 20 patients were included in this study. Baseline characteristics of included participants are presented in Table 1. Fourteen patients were female (70%), with an average age of 54.0 years (SD 14.4). Ten patients were randomized to the intervention arm and underwent PET/MR imaging. All PET/MRI scans were of diagnostic quality. In line with the inclusion criteria, the presumed diagnosis in most patients was unclear and the differential diagnosis comprised many potential pain generators. One exception is a patient in the control group in whom adverse reaction to metal was suspected and confirmed with additional MRI.

**Table 1:** Baseline characteristics of study participants

		Control (*n* = 10)	PET/MRI (*n* = 10)	Total (*n* = 20)
Female sex, no. (%)		6 (60%)	8 (80%)	14 (70%)
Age, year (SD)		49.0 (15.9)	58.9 (11.5)	54.0 (14.4)
Body mass index, kg/m^2^ (SD)		25.8 (3.3)	26.5 (6.2)	26.1 (4.8)
Pain side, no. (%)	Left	3 (30%)	6 (60%)	9 (45%)
	Right	6 (60%)	3 (30%)	9 (45%)
	Bilateral	1 (10%)	1 (10%)	2 (10%)

## Diagnostic work-up and clinical management: control group

Diagnostic and therapeutic procedures in the control group are shown in Table 2. The 10 participants in this group underwent a total of 11 diagnostic imaging procedures including MRI (*n* = 5), bone scintigraphy (*n* = 2), SPECT/CT (*n* = 1), CT (*n* = 1), repeat radiography (*n* = 1), and ultrasound (*n* = 1). Diagnostic procedures performed in this group included synovial fluid culture (*n* = 1), iliopsoas bursa aspiration and culture (*n* = 1), and steroid injection at the iliopsoas tendon (*n* = 1). At 12 months, a final expert diagnosis was established for 5 out of 10 patients, including adverse tissue reaction (*n* = 2), iliopsoas bursitis (*n* = 1), iliopsoas tendinopathy (*n* = 1), and anterior impingement (*n* = 1). For 5 participants in the control group following regular clinical diagnostic work-up, no definitive diagnosis explaining the pain symptoms was established at the end of the follow-up period.

**Table 2:** Overview of study participants randomized to the regular clinical care (control) group. Diagnosis at baseline, diagnostic and therapeutic procedures, and the final expert diagnosis based on all available information are listed

Patient	Symptoms	Prior imaging	Presumed diagnosis at baseline	Diagnostic and therapeutic procedures	Expert diagnosis
**1**	Right sided THR, pain in hip region associated with walking	Radiography	1. Iliopsoas tendinopathy 2. Iliopsoas bursitis 3. Rectus femoris tendinopathy	Synovial fluid culture – negative MRI: adverse reaction metal debris	Adverse reaction to metal debris
**2**	Bilateral THR, pain in both hips associated with hip flexion and adduction	Radiography	1. Anterior impingement 2. Iliopsoas tendinopathy	MRI: signs of iliopsoas bursitis	Iliopsoas bursitis
**3**	Right sided THR, continuous pain in hip region	Radiography	1. Gluteal tendinopathy 2. Nerve root compression	CT: no abnormalities	No diagnosis
**4**	Right sided THR, intermittent pain in upper leg and lateral hip	Radiography	Unknown	Radiography: no abnormalities	No diagnosis
**5**	Left sided THR, pain and functional disability hip flexion	Radiography, MRI	1. Iliopsoas tendinopathy 2. Iliopsoas bursitis	Ultrasound: no abnormalities	Iliopsoas tendinopathy
**6**	Left sided THR, pain in hip region associated with walking	Radiography	1. Gluteal tendinopathy 2. Facet arthropathy	Bone scintigraphy: no abnormalities	No diagnosis
**7**	Right sided THR, pain in posterior hip, radiating to knee and lower back	Radiography	Unknown	Bone scintigraphy: possible iliopsoas bursitis Iliopsoas bursa aspiration: negative for infection	No diagnosis
**8**	Left sided THR, pain in anterior hip. Concomitant low back pain	Radiography	1. Iliopsoas tendinopathy 2. Nerve root compression	MRI: facet arthropathy with possible nerve root compression	No diagnosis
**9**	Right sided THR, pain anterior hip radiating to lower leg, mainly with hip flexion	Radiography	1. Anterior impingement 2. Rectus femoris tendinopathy 3. Trochanteric bursitis	Steroid injection Iliopsoas tendon: no effect Bone scintigraphy: no abnormalities MRI: signs of anterior impingement	Anterior impingement
**10**	Right sided THR, pain and functional disability	Radiography	1. Adverse reaction to metal	MRI: adverse reaction to metal debris Prosthesis revision	Adverse reaction to metal debris

### Diagnostic work-up and clinical management: PET/MRI group

FDG PET MRI demonstrated multiple areas of increased uptake which could potentially be the cause of pain in all ten patients, including the peritrochanteric area (*n* = 5), periprosthetic area (*n* = 3), iliopsoas tendon (*n* = 3), medial gluteal tendon (*n* = 1), tensor fascia lata (*n* = 1), a capsular abnormality anteriorly (*n* = 1), possible lymph node/neuroma anterior soft tissues (*n* = 1), facet joints (*n* = 3), nerve roots (*n* = 2), and diffuse multiple joint uptake (*n* = 1). An overview of PET/MRI findings is provided in Table 3.

**Table 3:** Overview of sites of increased [^18^F]FDG uptake and pain generators according to expert diagnosis

Patient	Periprosthetic	Peritrochanteric	Anterior soft tissues	Lumbar spinal structures	Other	Pain generator according to expert diagnosis
**1**	No structural abnormality, uptake around neck, SUV_max_ 2.8		No structural abnormality, uptake located at capsule, SUV_max_ 4.9			Anterior soft tissues (Rectus femoris and iliopsoas)
**2**		Small bursa, SUV_max_ 3.3				No diagnosis
**3**		Small bursa, SUV_max_ 2.7		Facet arthropathy, SUV_max_ 2.7		Lumbar spinal structures (Facet joints)
4		No structural abnormality, uptake at medial gluteal insertion, SUV_max_ 2.4	No structural abnormality, uptake at iliopsoas, SUV_max_ 4.3	Mild disc bulging, uptake in extraforaminal nerve root, SUV_max_, 3.1		Anterior soft tissues (Iliopsoas impingement)
5		No structural abnormality, peritrochanteric uptake, SUV_max_ 3.1		Facet arthropathy, SUV_max_ 2.3		Perirochanteric (bursa/tendon); Lumbar spinal structures (Facet joints)
6			No structural abnormality, uptake at iliopsoas, SUV_max_ 3.3	Mild disc bulging, uptake in disc and neuroforamen, SUV_max_ 2.7		Anterior soft tissues (Iliopsoas impingement) Lumbar spinal structures (Nerve root);
7			No structural abnormality, uptake at iliopsoas, SUV_max_ 4.0	Facet arthropathy, SUV_max_ 4.5		Anterior soft tissues (Iliopsoas impingement)
8	Soft tissue edema, uptake around prosthesis neck, shaft, and lymph nodes, SUV_max_ 8.6				No structural abnormality, diffuse uptake in multiple joints, SUV_max_ 6.0	Periprosthetic (Prosthetic joint infection); Other (diffuse joint inflammation)
9	No structural abnormality, uptake around prosthesis neck (contralateral non-painful hip), SUV_max_ 5.9		Soft tissue structure possibly neural or lymphoid, SUV_max_ 2.6			No diagnosis
10		No structural abnormality, uptake at tensor fascia lata, SUV_max_ 4.3				Anterior soft tissues (cement fragment)

All subsequent procedures and clinical outcomes for these patients during the study period are summarized in Table 4. Additional diagnostic imaging was performed in 6 participants, including CT (*n* = 3), ultrasound (*n* = 2), bone scintigraphy (*n* = 2), and MRI (external hospital, *n* = 1). Synovial fluid culture was performed in 3 participants. Additional diagnostic procedures included steroid injections at the trochanteric bursa (*n* = 2), iliopsoas tendon (*n* = 2), rectus femoris tendon (*n* = 1), hip joint space (*n* = 1), and lumbar nerve root (*n* = 1). At the end of the study period, expert diagnoses based on all interventions and follow-up outcomes were anterior impingement (*n* = 4), radicular or referred pain from the spine due to nerve root compression or facet arthropathy (*n* = 3), trochanteric bursitis (*n* = 1), rectus femoris tendinopathy (*n* = 1), and prosthetic joint infection (*n* = 1). No definitive diagnosis could be established for 2 patients.

**Table 4:** Overview of study participants randomized to the PET/MRI group. Symptoms and presumed diagnosis at baseline, PET/MRI findings, diagnostic and therapeutic procedures, and the final expert diagnosis based on all available information are listed

Patient	Symptoms	Prior imaging	Presumed diagnosis at baseline	Combined PET/MRI findings	PET/MRI-based interventions	Outcome of PET/MRI based interventions	Procedures not based on PET/MRI	Effect of procedures	Long-term treatments and effects	Expert diagnosis	PET/MR value
**1**	Left sided THR, pain in groin region with hip flexion.	Radiography	1. Anterior impingement 2. Iliopsoas bursitis 3. Iliopsoas tendinopathy	1. Capsular abnormality possibly leading to impingement 2. Possible periprosthetic infection	1. Diagnostic ultrasound of the hip 2. Synovial fluid culture and bone scintigraphy	1. Capsular thickening 2. Negative	CT: slight tendinopathy indirect rectus femoris, followed by targeted injection (lidocaine and steroids, ultrasound guided)	Pain relief		Tendinopathy rectus femoris and iliopsoas	None
**2**	Right sided THR, pain in lateral hip region associated with pressure and walking	Radiography, MRI	1. Trochanteric bursitis 2. Iliopsoas bursitis	1. Potential trochanteric bursitis	1. Steroid injection trochanteric bursa (external hospital)	1. No effect				No diagnosis	None
**3**	Bilateral THR, right sided pain with deep hip flexion. Concomitant radicular pain right leg.	Radiography	1. Nerve root compression 2. rectus femoris tendinopathy 3. Iliopsoas tendinopathy	1. Active facet arthropathy at multiple levels 2.Potential trochanteric bursitis	1. No intervention performed 2. No intervention performed	1.- 2.-	Referral to neurosurgeon. Additional MRI lumbar spine (external hospital): facet joint arthropathy and discus bulging with slight central stenosis. No intervention performed			Facet arthropathy	Indicated new pain source
**4**	Bilateral THR, left sided pain in groin and medial leg, radiating to the lower leg and foot. Concomitant low back pain.	Radiography, MRI	1. Iliopsoas bursitis 2. Iliopsoas tendinopathy	1. Focal uptake in iliopsoas tendon, possibly due to impingement 2. Medial gluteal tendinopathy 3. Possible nerve root compression L4 due to disc herniation	1. CT to assess prosthesis cup 2. No intervention performed 3. Nerve root block L4 (left)	1. Anteversion acetabular cup leading to iliopsoas tendon impingement 2. - 3.No effect			Cup revision with pain relief	Iliopsoas impingement	Indicated new pain source
**5**	Right sided THR, continuous pain lateral hip, groin, and anterior side upper leg.	Radiography, MRI (pre-arthroplasty)	1. Facet joint arthropathy 2. Nerve root compression 3. Prosthesis infection	1. Bilateral peritrochanteric uptake without structural abnormality 2. Active facet joint arthropathy L4-L5 and L5-S1	1. Steroid injection trochanteric area (external hospital) 2. No intervention performed	1. Pain relief 2. -	Bone scintigraphy	Negative	Trochanteric area denervation with pain relief	Trochanteric pain, Facet joint arthropathy	Confirmed suspected pain source and indicated new pain source
**6**	Left sided THR, pain groin region referring to buttocks and upper leg. Concomitant radicular pain with known lumbar disc herniation.	Radiography, MRI	1. Iliopsoas bursitis 2. Iliopsoas tendinopathy 3. Nerve root compression	1. Nerve root compression due to disc herniation with uptake in nerve root L4 2. Focal uptake in iliopsoas tendon, possibly due to impingement	1. No intervention performed 2. Steroid injection iliopsoas tendon (external hospital)	1. - 2. No effect			1. Surgical discectomy for low back pain with partial pain relief 2. Prosthesis revision	Nerve root compression due to disc herniation, iliopsoas impingement	Confirmed suspected pain source and indicated new pain source
**7**	Bilateral THR, with pain both sides. Right sided pain is more pronounced, located in the groin and buttocks.	Radiography	1. Iliopsoas bursitis 2. Iliopsoas tendinopathy 3. Anterior impingement	1. Active facet arthropathy L3-L4 2. (iliopsoas impingement initially missed due to masking by physiological uptake around implant)	1. No intervention performed 2. No intervention performed		1.- 2. Injection iliopsoas tendon right side (ultrasound guided)	1.- 2. Partial pain relief after injection	Cup revision resulting in pain relief	Iliopsoas impingement	Confirmed suspected pain source
**8**	Left sided THR, continuous pain in hip region, exacerbates with hip flexion	Radiography, MRI	1. Iliopsoas tendinopathy 2. Iliopsoas bursitis 3. Rectus femoris tendinopathy	1. Prosthetic joint infection 2. Diffuse joint inflammation	1. Synovial fluid culture 2. No intervention performed	1. Positive 2.-			1. Prosthesis revision with pain relief	1. Prosthetic joint infection 2. Medication mediated diffuse joint inflammation	Indicated new pain source Indicated systemic disease
**9**	Bilateral THR, left sided pain in hip and upper leg	Radiography	1. Gluteal tendinopathy 2. Trochanteric bursitis	1. Possible neuroma or lymphadenopathy anterior in the hip 2. Moderate synovitis 3. Rectus femoris tendinopathy	1. Ultrasound guided biopsy 2. Synovial fluid culture steroid injection to assess pain source	1. Negative 2. Negative for infection, no effect of steroid injection			Pericapsular nerve root block with pain relief	No diagnosis	Excluded suspected pain source
**10**	Bilateral THR, left sided pain lateral hip and upper leg	Radiography	1. Iliopsoas tendinopathy 2. Trochanteric bursitis 3. Gluteal tendinopathy	1. Peritrochanteric uptake without structural abnormality extending to tensor fascia lata	1. No intervention performed		Synovial fluid cullture left hip CT hip showing possible anterior impingement and loose cement fragment anterior to joint, followed by injection of anaesthetic at fragment location (ultrasound guided)	Negative for infection No effect of injection		Anterior impingement due to loose cement fragment	None

PET/MRI identified 5 new pain sources corresponding with the final expert diagnosis (two times iliopsoas impingement, one facet arthropathy, one trochanteric bursitis, and one prosthetic joint infection). PET/MRI also confirmed 3 suspected pain sources (one nerve root compression, one facet joint arthropathy, and one iliopsoas impingement), and excluded one initially suspected pain source (trochanteric bursitis). This includes two patients where both a suspected diagnosis was confirmed and a second new pain source was identified. PET/MRI contributed to the diagnosis in seven patients. In one patient, in addition to contributing to the diagnosis at the region of pain, PET/MRI also identified systemic uptake of multiple joints, consistent with systemic disease, which correlated with symptoms that developed at a later stage.

### Insights into PET/MRI findings

**Fig. 1:**
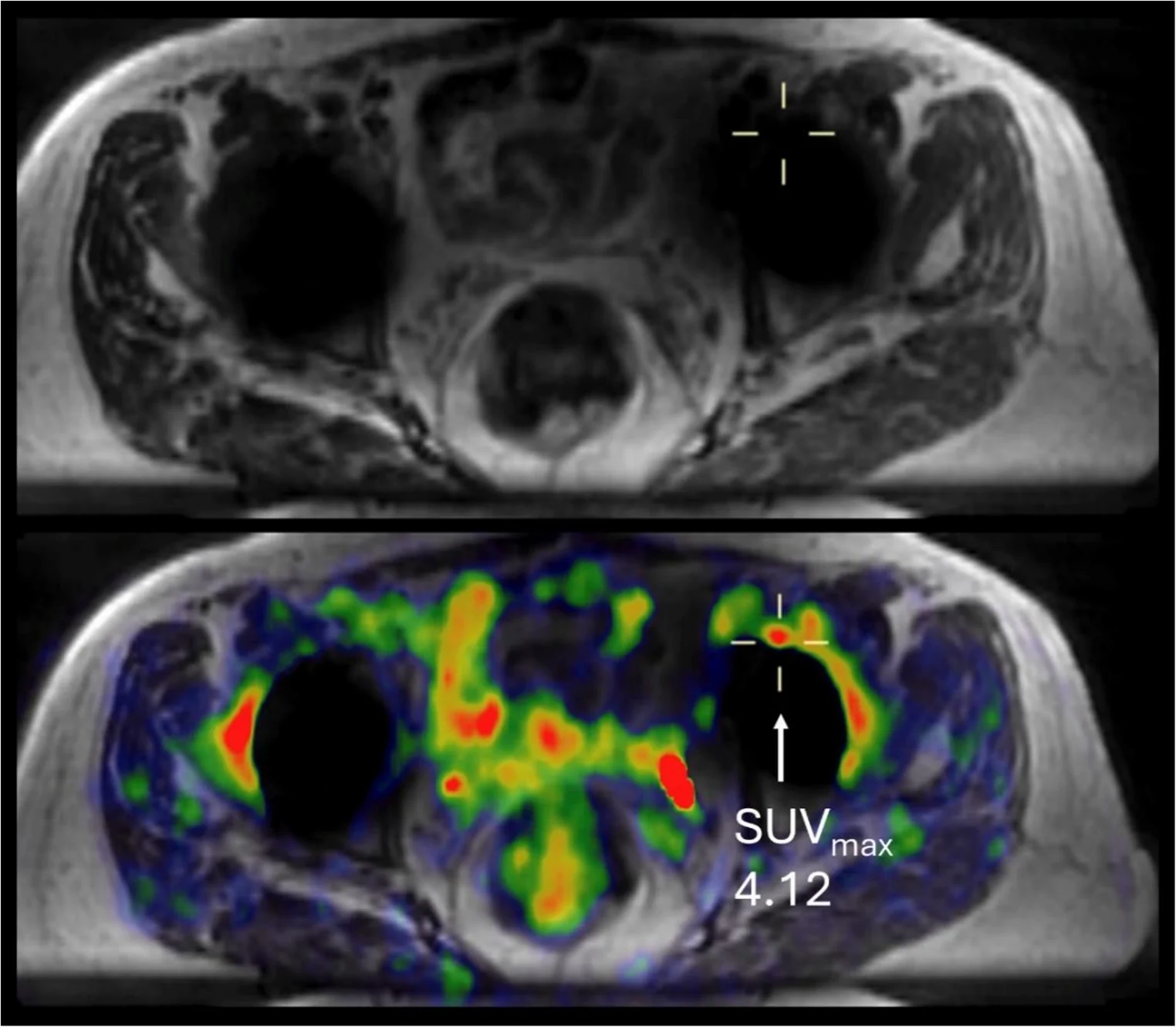
Example case of a patient with left-sided hip pain after bilateral hip replacement. [^18^F]FDG PET/MRI showed increased uptake in the psoas tendon, anterior to the prosthetic cup. PET also showed signs of mild nerve root compression due to slight disc herniation, which was the first treatment target given the impact of revision surgery. Targeted steroid injection at this nerve root had no effect on the pain. CT showed slight cup anteversion, and a repeat PET/MRI showed a similar uptake at the psoas tendon as the baseline PET/MRI convincing the orthopedic surgeon of the cause of pain. Patient then underwent surgical cup revision which relieved pain symptoms

**Fig. 2:**
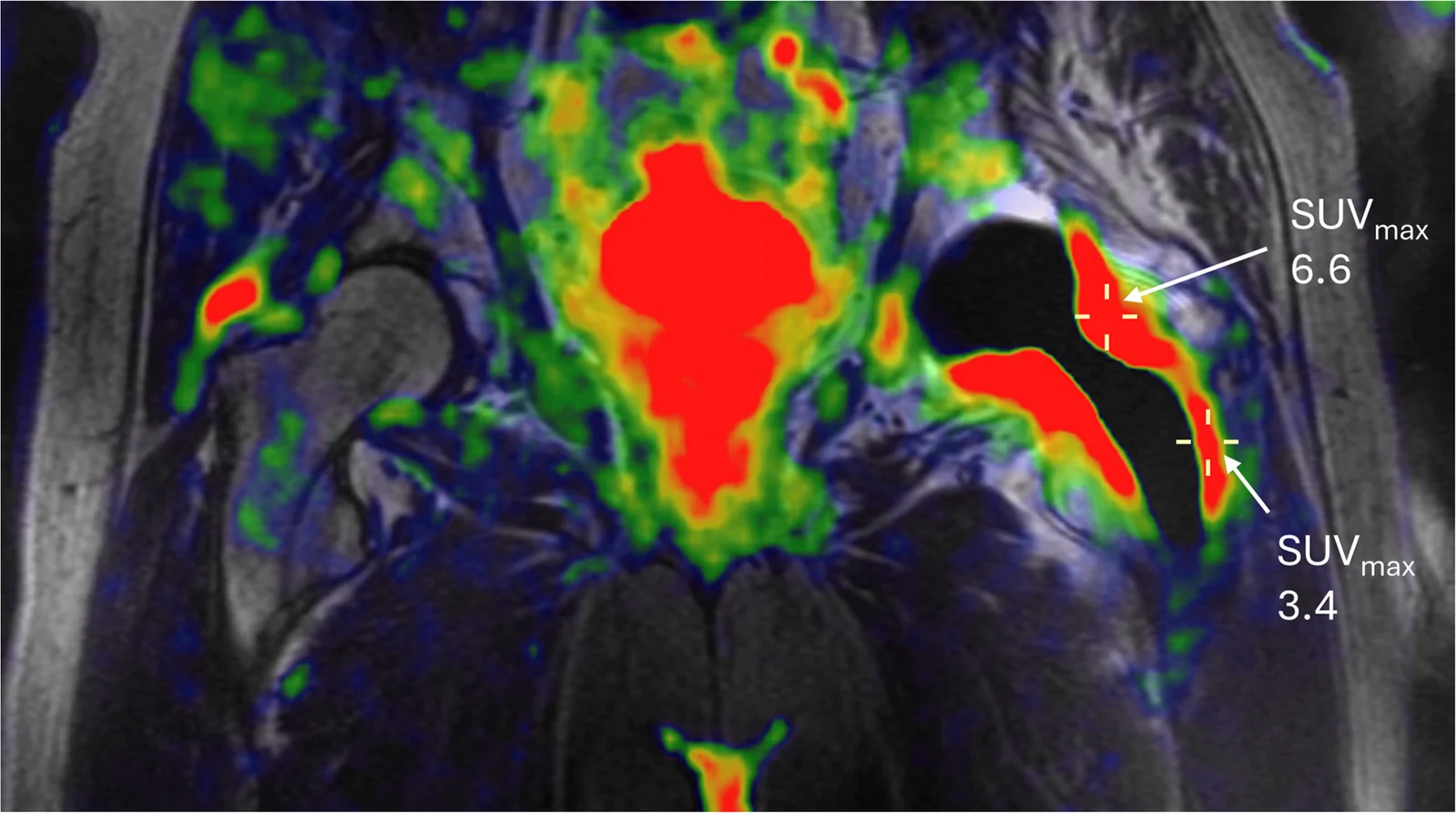
Example case of a patient with longstanding hip pain after left-sided total hip replacement. Radiography and bone scan showed no signs of infection. [^18^F]FDG PET/MRI shows increased uptake around the prosthesis neck, proximal shaft, and inguinal lymph nodes, suspicious for infection. Synovial fluid culture confirmed infection. Prosthesis revision followed and patient is now pain-free

Three patients had focal FDG uptake in the iliopsoas tendon at the level of the acetabular cup suggestive of iliopsoas tendinopathy due to anterior impingement. Example cases are shown in Figs. 1 and 2. In one of these patients anterior impingement was only retrospectively identified on PET/MRI, after initially being missed due to masking by high physiological periprosthetic FDG uptake. After subsequent clinical follow-up, anterior impingement was confirmed in these patients, either through pain relief after targeted injection, CT-confirmed cup malposition, or as a clinical diagnosis. All three patients underwent prosthesis revision which relieved the pain symptoms.

Five patients showed peritrochanteric FDG uptake. This may be related to greater trochanteric pain syndrome, formerly referred to as “trochanteric bursitis”, but it is most frequently caused by tendinopathy of the abductor muscles. Two patients demonstrated a small fluid filled bursa, one medial gluteal tendinopathy and two no structural abnormality. Two of these patients underwent peritrochanteric steroid injections at external hospitals, which resulted in pain relief in one patient and had no effect in the other patient. The patient with pain relief proceeded to bursal denervation and subsequent pain relief. The other patients did not undergo targeted treatment while other potential pain generators were prioritized.

Many patients (*n* = 7) showed signs of synovial inflammation on PET/MRI. In most cases, synovial FDG uptake was of low to moderate intensity and concentrated around the prosthesis neck, which is considered not be associated with periprosthetic infection [20]. One patient however showed intense inflammation along the prosthesis neck and the proximal shaft, likely associated with infection (see Fig. 2). Subsequent synovial fluid culture confirmed infection and this patient underwent surgical prosthesis revision. In 3 patients without suspicious uptake around the hip prosthesis, synovial fluid culture (*n* = 3) and/or bone scintigraphy (*n* = 2) were performed to rule out infection. All results were negative.

**Fig. 3:**
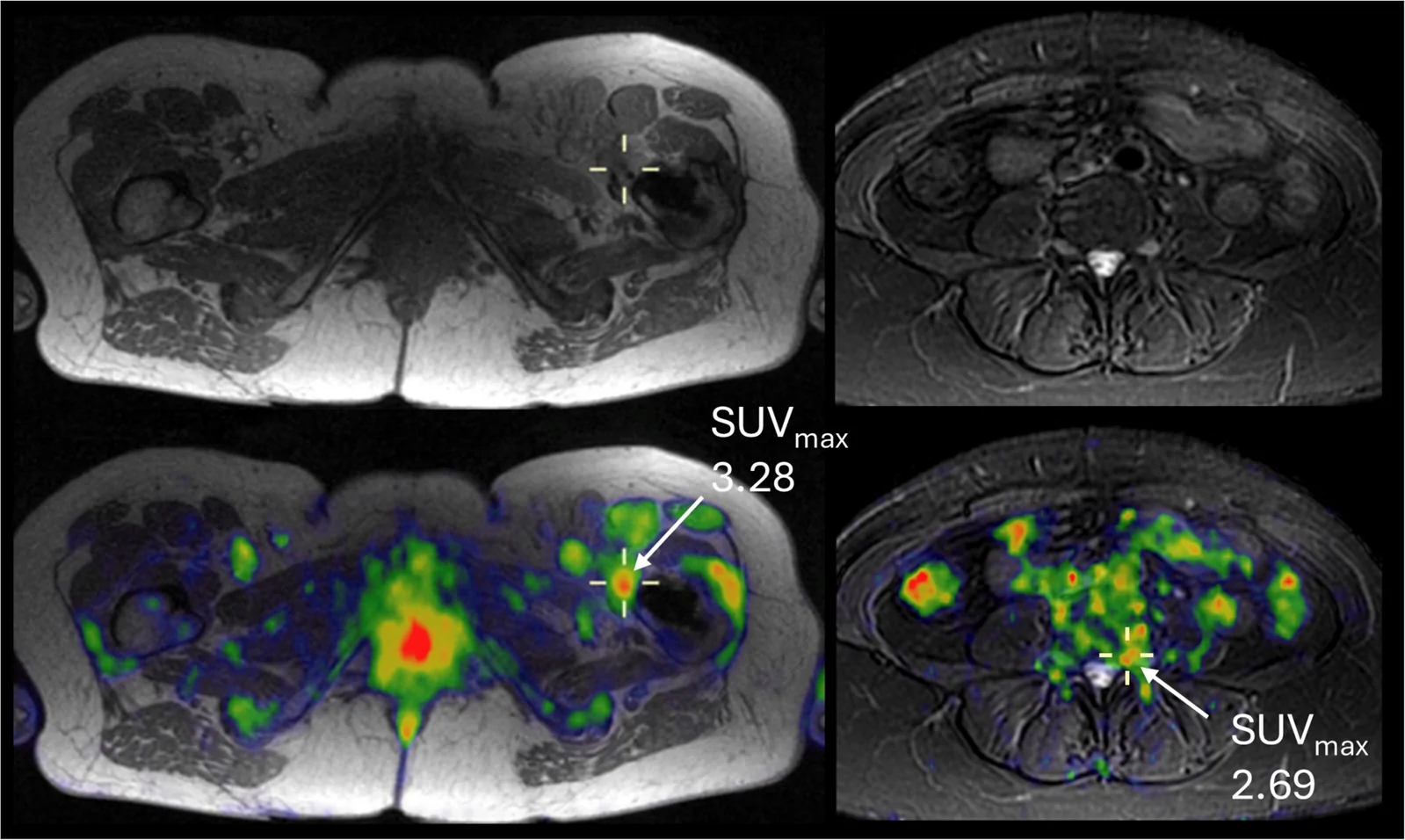
Example case of a patient with pain in the left hip and lower back. [^18^F]FDG PET/MRI showed psoas tendon uptake (shown left) and lumbar disc herniation with increased uptake (shown right). Surgical discectomy partially relieved pain, but severe pain symptoms still remained. This was followed by surgical revision of the prosthesis cup, which resulted in pain relief

Abnormalities of the lumbar spine were seen in 5 patients, potentially explaining patients’ symptoms though pain referral to the hip or as concomitant low back pain. Spinal abnormalities included active facet joint arthropathy in 3 patients and signs of nerve root compression in 2 patients. One patient underwent surgical discectomy to alleviate a compressed nerve root (see Fig. 3). Another patient underwent a nerve root block which ruled out nerve root compression as the pain source. Facet joint arthropathy was treated conservatively in these patients since targeted facet joint treatments are not reimbursed in the Dutch healthcare system. Less prevalent findings on PET/MRI included gluteal or rectus femoris tendinopathy and inflammation of the m. tensor fascia lata, most of which were treated conservatively. Diffuse uptake involving multiple joints was observed in one patient, consistent with systemic rheumatoid disease, which correlated with symptoms that developed at a later stage.

### Diagnostic confidence

Diagnostic confidence ratings were similar between baseline and after PET/MR, namely 7.8 (SD 1.6) and 8.0 (SD 1.3) respectively. The most likely diagnosis changed based on the PET/MR imaging results in 7 out of 10 patients, which corresponded with the final expert diagnosis in three cases.

### Patient reported outcomes

Baseline questionnaires were completed by all 10 participants in the intervention arm and 6 participants in the control arm. Follow-up at 6 months was completed by 9 intervention arm and 5 control arm participants, and 12-month follow-up was completed by 7 intervention and 4 control group participants. Mean NRS pain scores in the PET/MRI group were 6.1 (SD 2.3, range 4–10) at baseline and 5.2 (SD 2.2, range 2–8) at 6-months. One patient reported a decrease in pain score > 50% at 6 months, and 2 reported pain relief between 30% and 50%. Mean NRS for control group participants were 5.2 (SD 1.5, range 4–8) at baseline and 3.4 (SD 2.7, range 0–6) at 6-month follow-up. Two patients reported > 50% pain relief at 6 months, and three patients reported no or < 30% pain relief. All patient reported outcomes at baseline and during each follow-up timepoint are summarized in Table 5. GPE and treatment satisfaction ratings are graphically shown in supplementary figures S1 and S2.

**Table 5:** PROMS at baseline and at 1-, 3-, 6- and 12-month follow-up timepoints

		PET/MRI, mean (SD)	Control, mean (SD)
Average NRS Pain score	Baseline (*n* = 16)	6.1 (2.3)	5.2 (1.5)
	1 month (*n* = 12)	5.4 (1.7)	3.6 (2.5)
	3 months (*n* = 14)	5.1 (2.6)	5.0 (2.3)
	6 months (*n* = 14)	5.2 (2.2)	3.4 (2.7)
	12 months (*n* = 11)	4.1 (1.8)	1.8 (1.5)
Maximum NRS Pain score	Baseline	7.9 (1.4)	7.0 (1.1)
	1 month	7.1 (1.7)	4.6 (3.2)
	3 months	6.6 (2.5)	6.2 (2.9)
	6 months	6.6 (1.9)	4.4 (3.8)
	12 months	6.1 (1.7)	2.5 (1.3)
Oxford Hip Score	Baseline	21.0 (9.4)	29.3 (4.2)
	1 month	25.1 (8.8)	31.0 (4.5)
	3 months	25.0 (7.6)	30.2 (8.5)
	6 months	27.0 (7.9)	33.0 (7.7)
	12 months	27.7 (5.8)	39.0 (3.9)
Eq. 5D-value	Baseline	0.54 (0.34)	0.72 (0.09)
	1 month	0.70 (0.08)	0.75 (0.12)
	3 months	0.67 (0.06)	0.75 (0.15)
	6 months	0.68 (0.10)	0.77 (0.15)
	12 months	0.67 (0.09)	0.77 (0.11)
HADS Anxiety score	Baseline	5.9 (5.0)	3.8 (3.3)
	6 months	5.9 (4.2)	3.5 (2.1)
	12 months	4.6 (5.1)	3.0 (2.3)
HADS Depression score	Baseline	7.1 (4.7)	5.5 (4.1)
	6 months	7.0 (3.5)	7.5 (0.7)
	12 months	5.3 (4.4)	2.8 (1.7)

## Discussion

In this study, the diagnostic role of hybrid [^18^F]FDG PET/MRI in patients with chronic hip pain after THR was evaluated in real-life clinical practice. Our findings suggest that PET/MRI can identify abnormalities in the hip and lumbar spine region that may be associated with patients’ pain symptoms. PET/MRI contributed to the diagnostic process in most patients by identifying potential pain generators and guiding additional imaging or targeted interventions. A higher proportion of patients in the PET/MRI group received a diagnosis compared to those undergoing regular diagnostic work-up. Mean pain scores decreased in both groups, but between-group differences could not be statistically tested due to insufficient statistical power. Although FDG PET/MRI guided treatment often led to immediate positive effects, this was not fully reflected in the clinical outcomes at 6 and 12 months in this small study. Several factors may have contributed to these results, which are discussed in more detail in the following paragraphs.

The ability of PET/MRI to detect increased FDG uptake in regions corresponding to pain symptoms suggests that metabolic imaging may provide valuable insights into underlying pathophysiology, such as low-grade inflammation. Previously, mostly observational studies have indicated that [^18^F]FDG PET/MRI can identify pain generators, increase diagnostic confidence and subsequently influence management in patients with chronic low back pain [12–16]. Our study shows that [^18^F]FDG PET/MRI may also benefit patients with persistent pain after joint arthroplasty. PET/MRI led to more diagnoses compared to the regular diagnostic work-up, and subsequent targeted treatment led to positive clinical outcome in some patients, showing that PET/MRI is also able to accurately locate pain sources in the hip. Another possible advantage of PET/MRI reflected by our findings is its sensitivity to alternative pain sources that may cause referred pain, such as spinal abnormalities, as well as its ability to detect systemic disease in cases of multi-joint involvement.

Despite the promising findings, implementation of a novel diagnostic modality is challenging in real-life clinical practice and many important considerations should be addressed in future studies. Firstly, the interpretation of PET/MRI scans can be challenging. SUVs of potentially clinically relevant abnormalities may approximate those of physiological background activity (e.g., bone marrow), thereby increasing the risk of both false-positive and false-negative findings. As the use of FDG PET/MRI is novel for this clinical indication and the physiological uptake can be substantial in certain areas around hip prostheses, accurate interpretation remains dependent on reader experience and clinical correlation. Referring orthopedic surgeons also required time to develop confidence and incorporate the new insights into their clinical workflow. It was noted that during this study, findings from PET/MRI were not always directly acted upon and findings were not always consistently translated into targeted treatment strategies. Chronic pain after THR is often multifactorial, and even when a primary pain generator is identified and treated, symptoms may not fully resolve until all contributing sources of pain are addressed. It is therefore possible that targeted treatments are effective, even when the treatment effect is not fully reflected in patient reported pain scores. It is also important to note that some treatment decisions, particularly those involving invasive procedures such as revision surgery, can carry significant risks. Treating physicians therefore tend to be cautious and aim for a high level of confidence in the presence of structural pathology before opting for surgical intervention. Treatment choices likely also depend on cultural and healthcare system differences. Real-life implementation within the Dutch healthcare system including referrals to community hospitals introduced additional challenges. For example, not all diagnostic injections were confirmed to be performed under ultrasound guidance, limiting confirmation of procedural accuracy. In the Netherlands, cost-effectiveness is highly important, leaving clinicians sometimes reluctant with targeted treatments that are not reimbursed. Similarly, patients reporting with chronic pain do not routinely undergo CT or MR imaging, and therefore most patients in our cohort had not undergone prior imaging before trial participation. As such, findings from our study should not be generalized to other healthcare systems. Pre-trial MRI in our cohort likely would have identified the two patients in the control group with an adverse reaction to metal debris. Finally, it is highly likely that the short-duration effects of some treatments were missed in the patient reported outcomes due to the relatively long follow-up intervals. For example, the effect of steroid and anaesthetic injections generally only lasts for several months and thus not always accurately captured with the follow-up timepoints in our study. Conversely, longer follow-up may be needed to capture long-term effects of certain interventions including any delays in treatment. Consequently, the patient reported outcomes may not accurately reflect the true effectiveness of PET/MRI in this patient group.

Strengths of this study include its randomized design, the prospective collection of both diagnostic outcomes and patient-reported outcomes and the real-life clinical practice implementation. However, several limitations must be acknowledged. First, the number of inclusions and the response rate to questionnaires with PROMs were lower than expected, resulting in insufficient statistical power to sufficiently evaluate between-group differences in PROMs. Questionnaire response rates were also lower in the control group, possibly due to participants’ disappointment regarding group allocation and potentially inducing some attrition bias for PROMs. Moreover, it has become evident that clinical outcomes are influenced by many factors as outlined above; therefore, we focused on the treatment effect after targeted intervention, which was jointly evaluated by the clinician and patient. This measure more accurately reflects the immediate impact of PET/MRI targeted treatment. Second, our study design did not allow for study participants and treating physicians to be blinded to group allocation, which may have induced some bias. Next, the pragmatic design of our study reflects daily clinical practice, but the lack of standardized work-up may have introduced variability in the control group. Nevertheless, we believe our control group is an acceptable reflection of current clinical practice and our study outcomes should be interpreted in this context. Technical limitations of PET/MRI in patients with metal implants, such as insufficient attenuation correction and residual metal artifacts, may also have obscured potential abnormalities and influenced diagnostic outcomes. Although all scans were of diagnostic quality, future technological advancements may improve interpretability. PET/MRI also remains limited in availability, while PET/CT is more widely accessible. In centers without integrated PET/MRI systems, this limitation may be mitigated by combining PET data acquired from PET/CT with MRI data using software-based image fusion. Lastly, while FDG has shown potential in chronic pain, it does not capture all underlying pain processes. Exploration of alternative PET tracers that target other physiological processes in the context of chronic musculoskeletal pain, such as sodium fluoride or fibroblast activation protein inhibitor, may be of interest for future studies.

To the best of our knowledge, this is the first study investigating [^18^F]FDG PET/MRI as a diagnostic imaging modality for patients with persisting hip pain after THR. Our findings demonstrate the potential of PET/MRI to reveal new pain sources or confirm suspected pain sources and exclude initially suspected pain sources, which could guide and strengthen clinicians in their treatment decisions. Further research should include larger, well-controlled multi-center trials to confirm our findings. Another important factor to explore is standardizing treatment pathways based on PET/MRI results, which potentially could lead to faster time-to-diagnosis and improved clinical outcomes.

## Conclusion

Initial results indicate the potential of [^18^F]FDG PET/MRI for identifying pain sources in patients with diagnostically challenging chronic hip pain after THR by revealing new pain sources and excluding initially suspected pain sources. Further research regarding clinical outcomes is warranted in a larger cohort with standardized treatment strategies following PET/MRI findings.

## Supplementary Information

Below is the link to the electronic supplementary material.

Supplementary Material 1 (DOCX 718 KB)

## Data Availability

The datasets generated during and/or analysed during the current study are available from the corresponding author on reasonable request.
