# Supplementary material for: The potential of [18F]FDG PET/MRI for identifying pain sources in patients with diagnostically challenging chronic hip pain after total hip replacement
Source: Eur J Nucl Med Mol Imaging. 2026 Aug 22;53(13):7729–42. doi: 10.1007/s00259-026-08142-4 (PMC13727810; doi:10.1007/s00259-026-08142-4)
Supplement: Supplementary file 1 [file 259_2026_8142_MOESM1_ESM.docx]

Supplementary information to:

**The potential of [^18^F]FDG PET/MRI for identifying pain sources in patients with diagnostically challenging chronic hip pain after THR**

*Table S1. MRI pulse sequence parameters for hybrid PET/MRI of the painful hip after total hip replacement.*

| Sequence | Imaging plane | TR/TE (ms) | Flip Angle | Acquisition matrix | FOV (mm) | Slice Thickness (mm) | NEX |
| --- | --- | --- | --- | --- | --- | --- | --- |
| 3D LAVA T1 Dixon | Axial | 5.1/1.8 | 15 | 512 x 512 | 420 x 420 | 2.0 | 0.69 |
| 2D FSE T2 Dixon | Axial | 9608/69.8 | 160 | 512 x 512 | 420 x 420 | 4.0 | 3 |
| 3D T1 MAVRIC SL | Coronal | 375/6.7 | 90 | 512 x 512 | 500 x 500 | 2.0 | 0.5 |
| 3D MAVRIC SL Fluid | Coronal | 7600/5.8 | 75 | 512 x 512 | 500 x 500 | 2.4 | 0.5 |
| MSDE-CUBE T2 | Coronal | 2502/78.8 | 90 | 512 x 512 | 500 x 500 | 1.2 | 3 |
| 3D T1 MAVRIC SL^†^ | Coronal | 375/6.7 | 90 | 512 x 512 | 500 x 500 | 2.0 | 0.5 |

^†^*After injection of gadolinium-based contrast agent. TR = Repetition time; TE = Echo time; FOV = Field of view; NEX = Number of Averages; LAVA = Liver Acquisition with Volume Acceleration; FSE = Fast Spin Echo; MAVRIC= Multi-Acquisition Variable-Resonance Image Combination; MSDE = motion-sensitized driven-equilibrium.*

*Figure S1. Clustered self-rated perceived treatment effect for PET/MRI and control group participants. Questionnaires were taken at baseline (BL), 1 month (M1), 3 months (M3), 6 months (M6) and 12 months (M12).*

*Figure S2. Clustered self-rated treatment satisfaction for PET/MRI and control group participants. Questionnaires were taken at baseline (BL), 1 month (M1), 3 months (M3), 6 months (M6) and 12 months (M12).*
